# Identifying Stereoisomers by *ab-initio* Calculation of Secondary Isotope Shifts on NMR Chemical Shieldings

**DOI:** 10.3390/molecules19045301

**Published:** 2014-04-23

**Authors:** Karl-Heinz Böhm, Klaus Banert, Alexander A. Auer

**Affiliations:** 1 Max-Planck-Institute for Chemical Energy Conversion, Stiftstraße 34–36, D-45470 Mülheim an der Ruhr, Germany; E-Mail: karl-heinz.boehm@cec.mpg.de; 2 Institut für Chemie, Technische Universität Chemnitz, Straße der Nationen 62, D-09111 Chemnitz, Germany; E-Mail: klaus.banert@chemie.tu-chemnitz.de

**Keywords:** secondary isotope effects, NMR chemical shieldings, zero point vibrational effects, electronic structure calculations, *ab-initio* calculations

## Abstract

We present *ab-initio* calculations of secondary isotope effects on NMR chemical shieldings. The change of the NMR chemical shift of a certain nucleus that is observed if another nucleus is replaced by a different isotope can be calculated by computing vibrational corrections on the NMR parameters using electronic structure methods. We demonstrate that the accuracy of the computational results is sufficient to even distinguish different conformers. For this purpose, benchmark calculations for fluoro(2-^2^H)ethane in gauche and antiperiplanar conformation are carried out at the HF, MP2 and CCSD(T) level of theory using basis sets ranging from double- to quadruple-zeta quality. The methodology is applied to the secondary isotope shifts for 2-fluoronorbornane in order to resolve an ambiguity in the literature on the assignment of *endo*- and *exo*-2-fluoronorbornanes with deuterium substituents in *endo*-3 and *exo*-3 positions, also yielding insight into mechanistic details of the corresponding synthesis.

## 1. Introduction

Quantum chemical calculations provide a powerful tool to investigate molecular properties and therefore support the interpretation of experimental findings. This applies especially to NMR chemical shieldings, as these contain a wealth of structural information that is, however, only indirectly related to the molecular structure itself. Nevertheless, in the interplay of theory and experiment, detailed structural information can be obtained by computing and comparing the molecular structure and the associated NMR parameters. In recent years, several studies have demonstrated that NMR chemical shifts can be calculated quantitatively using highly accurate methods [1–3]. For ^19^F chemical shieldings, for example, which range from about −200 to +500 ppm, Harding *et al.* presented a detailed benchmark study [4]: For the level of electron correlation they find, that compared to CCSD(T), HF leads to 10–20 ppm deviation and MP2 leads to about 5 ppm deviation. With respect to the shieldings obtained at the CCSD(T)/13s9p4d3f level of theory, basis set errors of about 10 ppm occur when the tzp basis is used, about 3 ppm when qz2p is used, and less than 1 ppm when the pz3d2f basis set is used. Furthermore, vibrational corrections range between −5 and −21 ppm while temperature corrections are typically one order of magnitude smaller (−0.2 to −2.9 ppm) and are treated in similar quality on HF and MP2 level of theory. In related studies, similar observations were made for ^13^C, ^17^O and ^15^N chemical shieldings [1,2,5].

The possibility to calculate zero-point vibrational effects and temperature corrections on NMR chemical shieldings also allows to predict secondary isotope shifts on chemical shieldings Δ*σ* via Δ*σ* = *σ_l_* − *σ_h_*. These shifts occur when a lighter nucleus *l* different from the resonant one is substituted by a heavier isotope *h* [6]. If this effect is calculated using quantum chemical methods, it has to be noted that the electronic Schrödinger equation in the framework of the Born–Oppenheimer approximation is independent of the nuclear masses. Thus, one has to consider the nuclear Schrödinger equation, where isotope effects occur as vibrational and rotational effects [2,7]. Since the average positions of the nuclei do not change under change of masses of the nuclei in the harmonic-oscillator model, at least a cubic force field model has to be considered [8–10] when isotope effects on chemical shieldings are computed.

In order to calculate isotope shifts, several theoretical approaches have been propsed in the past [11–13]. In this work, we apply vibrational perturbation theory of second order (VPT2 [14]), which allows to include vibrational, temperature, and rotational corrections. In the framework of VPT2 the vibrationally averaged isotropic shieldings are obtained as:

(1) $$\sigma_{0}=\sigma (r_{e})+\sum_{r}{\left(\frac{\partial \sigma }{\partial Q_{r}}\right)}_{Q_{r}=0}\langle Q_{r}\rangle +\frac{1}{2}\sum_{r,s}{\left(\frac{\partial^{2}\sigma }{\partial Q_{r}\partial Q_{s}}\right)}_{Q_{r},Q_{s}=0}\langle Q_{r}Q_{s}\rangle$$

On the right hand side appears the isotropic shielding *σ*(*r_e_*) at the equilibrium geometry *r_e_*, the sum of all derivatives of the shielding with respect to the normal coordinates ${\left(\frac{\partial \sigma }{\partial Q_{r}}\right)}_{Q_{r}=0}$ times the mean displacements of the nuclei 〈*Q_r_*〉 and the sum of all second derivatives ${\left(\frac{\partial^{2}\sigma }{\partial Q_{r}\partial Q_{s}}\right)}_{Q_{r},Q_{s}=0}$ times the mean-square displacements of the nuclei 〈*Q_r_Q_s_*〉 with respect to the normal coordinates. The mean and the mean-square displacements are given by the following equations:

(2) $$\langle Q_{r}\rangle =-\frac{\hbar }{4\omega_{r}^{2}}\left[\sum_{s}\frac{k_{rss}}{\omega_{s}}\coth\left(\frac{\hbar \omega_{s}}{2k_{B}T}\right)-2k_{B}T\sum_{\alpha }\frac{a_{r}^{\left(\alpha \alpha \right)}}{I_{\alpha \alpha }}\right]$$

(3) $$\langle Q_{r}Q_{s}\rangle =\delta_{rs}\frac{\hbar }{2\omega_{r}}\coth\left(\frac{\hbar \omega_{r}}{2k_{B}T}\right)$$

Here *ω_r_* denotes the vibrational frequencies, *k_rss_* the cubic force constant, *T* the temperature, *I_αα_* the momentum of inertia with respect to the *α*'th principal axis and $a_{r}^{\left(\alpha \alpha \right)}$ the first coefficient in the expansion of *I_αα_* with respect to the normal coordinates.

While calculations of secondary isotope shifts are far from routine applications of quantum chemical methods, previous work has shown that for small molecular systems these calculations can yield quantitative results, provided the correct treatment of vibrational effects on nuclear shieldings is used [3]. The focus of this study was on the secondary isotope effects on methanol for which it was not only demonstrated that highly accurate ab-initio methods yield quantitative results, but that the secondary isotope shifts could even be reproduced in acceptable agreement with experimental values at a very cost effective level of theory (namely Hartree–Fock) owing to systematic cancellation of errors. In this work, we investigate whether and how the calculation of secondary isotope shifts on NMR chemical shieldings can be applied to identify different stereoisomers using electronic structure methods. After discussing computational details, we present a benchmark study for fluroethane, a compound that is small enough to carry out high level CCSD(T) calculations. Note that here, we focus on quantifying the different sources of error from method, basis set and treatment of virbrational- and temperature effects by comparison to the highest available level, rather than by comparison to experimental values.

As an example, for which quantum chemical calculations can actually resolve ambiguities in the literature, we would like to revisit in the second part of this work one of the first studies in which various effects on secondary isotope shifts on NMR chemical shieldings were systematically investigated. Lambert *et al.* [15] experimentally investigated secondary isotope effects on NMR chemical shieldings on *endo*-2-fluoronorbornanes and fluorocyclohexanes in order to identify major influences on secondary isotope shifts. Especially the results for the *endo*-2-fluoronorbornanes are interesting as our results [16] suggest that in contrast to what has been assumed by Lambert *et al.*, the synthesis chosen does not lead to the desired *endo*-2-fluoronorbornanes. Instead, mixtures of *exo*-2-fluoronorbornanes are obtained with deuterium nuclei bound to the 3- and 7-carbon atom.

## 2. Computational Details

All calculations were carried out using the CFOUR program package [17] where vibrational averaged nuclear shieldings are calculated as follows:

Given the molecular geometry as obtained from a well converged geometry optimization, the nuclear shieldings and the harmonic force field are calculated. In order to compute the first and second derivative of the nuclear shieldings with respect to the normal coordinates as well as the cubic force field numerically, the nuclei of the molecule have to be displaced along the normal coordinates and at each geometry the nuclear shieldings and the quadratic force field are determined. Subsequently, the vibrational averaged isotropic shieldings are obtained using Equations (1)–(3) [14].

For systems with many degrees of freedom, this procedure leads to a large number of calculations owing to the number of vibrational modes, especially in case of unsymmetrical molecules like 2-fluoronorbornanes, where 204 single calculations per compound are required.

As the calculation of the cubic force field and the nuclear shielding are done in two separate calculations, they can be carried out at two different levels of theory. This is advisable as for reliable results for the force field, a different level of theory is required than for NMR chemical shieldings [2,4]. In the following, this was done for benchmark calculations on fluoroethane, where the cubic force field was calculated using the HF method with tz2p and qz2p basis sets [18] and at the MP2/cc-pVTZ level of theory [19], while the nuclear shieldings were calculated using the HF [20,21], MP2 [22,23], and CCSD(T) [24] methods with tz2p and qz2p basis sets. The calculations on the much larger fluoronorbornanes presented in the subsequent section, where carried out at the HF/tz2p level of theory. All calculations of nuclear shieldings were carried out using gauge including atomic orbitals (GIAOs) [20,25,26].

It should be noted that the choice of the stepsize of displacement of the nuclei along the normal coordinates in the numerical third derivative procedure introduces an error of about 10^−3^ ppm for the ^13^C isotope shifts, while for the ^19^F isotope shifts this error is one order of magnitude larger. In the case of small frequencies (*ω* ≤ 100 cm^−1^) the contributions to the vibrational correction are overestimated, and contributions with (*ω* ≤ 200 cm^−1^) are excluded [5,27]. When introducing the nuclear mass of ^13^C_5_ in *endo*-2-fluoro(*exo*-3-^2^H)norbornane, the value of the ^13^C secondary isotope shift on C_5_ is the same as if the nuclear mass of ^12^C is used. Therefore, all calculations were done using the mass of ^12^C for all carbon nuclei, which has been shown to have very little effect on the calculated values as in contrast to the substitution of ^1^H by ^2^H (100% mass increase), the mass difference between ^12^C and ^13^C is only 8%.

### Benchmark Calculations of Secondary Isotope Shifts on Fluoroethane

If species are to be identified by their secondary isotope shifts, the relative differences between the values of different compounds are important as well as the absolute values for each species. At the appropriate level of theory, to allow a reasonable assignment, the corresponding error due to method and basis set needs to be lower than the relative isotope shifts.

**Figure 1.**
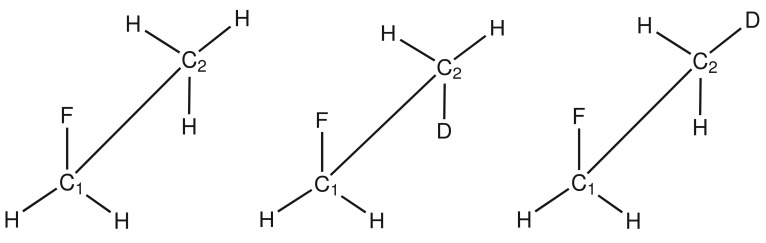
Fluoroethane without deuterium label (**left**), with deuterium in antiperiplanar conformation (**center**) and with deuterium in gauche conformation (**right**) with respect to the fluorine atom.

To estimate the errors of different computational methods, benchmark calculations were carried out for fluoroethane, which constitutes a small model system for which high-level calculations are feasible. The calculated ^13^C secondary isotope shifts on chemical shieldings of carbon nuclei on the 1 and 2 position and the secondary isotope shifts on chemical shieldings of the ^19^F nucleus are given in Table 1. In fluoroethane, deuterium is located in the gauche and antiperiplanar conformations relative to the fluorine atom (Figure 1).

All isotope shifts are given for T = 0 K and T = 300 K to assess the influence of the temperature correction on the isotope shifts. Temperature effects on secondary isotope shifts can be sizeable, as the inclusion of temperature corrections lowers isotope shifts on fluorine by about 20% at all levels of theory. For carbon atoms, on the other hand, the inclusion of temperature corrections leads to larger isotope effects, especially in the case of C_1_ with deuterium in the antiperiplanar conformation.

Comparison of the isotope shifts obtained at HF/tz2p with those obtained at HF/qz2p level of theory shows that deviations of about 10% arise for the fluorine atom while they amount to about 7% for C_1_ and amount to less than 1% for C_2_.

While at CCSD(T)/qz2p + MP2/cc-pVTZ level of theory correlation effects are incorporated, at HF/tz2p level of theory this is not the case. This leads to an underestimation of the ^19^F secondary isotope shifts of at most 0.057 ppm and 0.050 ppm on average at the HF/tz2p level of theory. The isotope effects at C_1_ are underestimated by at most 0.007 ppm and 0.006 ppm on average and at C_2_ they are at most 0.013 ppm larger and on average they are about 0.010 ppm larger than at the CCSD(T)/qz2p + MP2/cc-pVTZ level of theory. The values at the CCSD(T)/qz2p + MP2/cc-pVTZ level show only little deviations from the values given at the MP2/qz2p + MP2/cc-pVTZ level of theory, which leads to the conclusion that, already at the MP2/qz2p level, correlation effects are sufficiently taken into account.

In order to assess the main sources of error at the most cost-effcient level of theory, namely HF/tz2p, Table 1 also contains results from calculations, for which either the chemical shifts or the force field has been calculated at the MP2 level of theory. While generally both influences are of equal importance, there is a slight trend visible, that the calculation of the chemical shifts at the MP2 level of theory improves the results more than if only the force field is calcuated at the MP2 level of theory, which in some cases leads to an overestimation of the secondary isotop effects with respect to the full MP2 result.

Next, we turn to the errors in the difference between isotope effects on the shieldings of the gauche and antiperiplanar conformations, which is the decisive quantity if the different conformers are to be distinguished by the computed values. As the errors due to basis set effects and level of theory are very systematic, a favourable error compensation can be observed, such that the errors for the relative values are of the same order or less than for the secondary isotope shifts themselves. While the error in the chemical shifts is in the order of 10 ppm [29], the error in the vibrational corrections is in the order of less than 0.1 ppm while the error in the secondary isotope effects is in the order of 0.01 ppm. Thus, similar differences on all levels of theory (Table 1) are obtained. While the inclusion of the temperature correction leads to almost the same difference in the carbon isotope shifts of both conformations, the difference in the fluorine isotope shift decreases by about 0.04 ppm at all levels of theory. As a consequence, relative values of secondary isotope shifts can be calculated qualitatively even at the HF/tz2p level of theory, although the error on the absolute value of the isotope shifts might be fairly large. This is in agreement with previous results and also enables us to carry out calculations for much larger molecular systems, for which the computational effort for calculations at the CCSD(T) level of theory, for example, would be prohibitive.

## 3. Configurational Analysis of *endo*-2-Fluoronorbornanes—Challenging Literature

**Figure 2.**
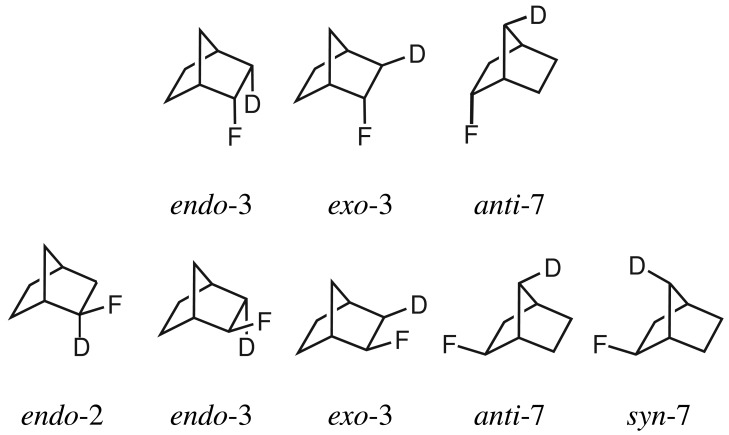
*endo*-2-Fluoronorbornane (**top**) and *exo*-2-fluoronorbornane (**bottom**) with deuterium labels in various positions.

An early example for exploring dependencies of various parameters on secondary isotope effects on ^19^F chemical shieldings was conducted by Lambert *et al.* [15]. Aiming to understand the dependence on the distance and the dihedral angle between substituted and resonating nuclei as well as on the number of substituted nuclei on secondary isotope shifts, several compounds were studied including 2-fluoronorbornanes, where ^1^H in the *endo*-3 and *exo*-3 positions were substituted by deuterium (Figure 2). It was claimed that the correspondingly deuterated *exo*-2-norbornanoles react via a S*_N_*2 mechanism under inversion to *endo*-2-fluoronorbornanes. However, the postulated S*_N_*2 mechanism is questionable and the reaction may proceed via a S*_N_*1 mechanism [16,27].

It is well known that inverting S*_N_*2 displacements at 2-norbornyl substrates proceed very slowly because of substancial steric hindrance. This is quite different to analogous reactions of monocyclic starting compounds such as cyclopentyl or cyclohexyl derivatives [30]. In the case of *exo*-2-norbornyl substrates, the comprehensively investigated S*_N_*1 reactions are reported to be unusually fast if compared to similar transformations of other compounds. Such competing unimolecular ionization reactions lead to equilibration of a single deuterium label and to exclusive formation *exo*-2-norbornyl substitution products via an intermediate ambident nonclassical carbenium ion or, alternatively, via very rapid Wagner–Meerwein rearrangement of classical carbocations [31].

The secondary isotope shifts of the molecules which were claimed by Lambert *et al.* and of the products of a S*_N_*1 substitution, can be calculated using quantum chemical methods to assess whether the the values of the configurations are correctly assigned in the work of Lambert *et al*.

In Table 2, the calculated secondary isotope shifts of the *endo*-2-fluoronorbornanes with deuterium in the *endo*-3, *exo*-3 and *anti*-7 position are given. While deuteration at the *endo*-3 position leads to an isotope shift of −0.291 ppm, deuteration at the *exo*-3 position leads to an isotope shift of −0.108 ppm. Therefore, the compounds should be distinguishable with the help of the computational results, due to the significant differences in the isotope shifts. Note that from the previous section, uncertainties of less than 0.020 ppm can be anticipated for the calculated relative differences in the isotope shifts. In contrast to the calculated values, the secondary isotope shifts obtained by Lambert *et al.* [15] amount to −0.15 ± 0.06 ppm for the *endo*-3-deutero compound and −0.40 ± 0.06 ppm for the *exo*-3-deutero compound. Within the given computational and experimental uncertainties these results do not coincide.

**Figure 3.**
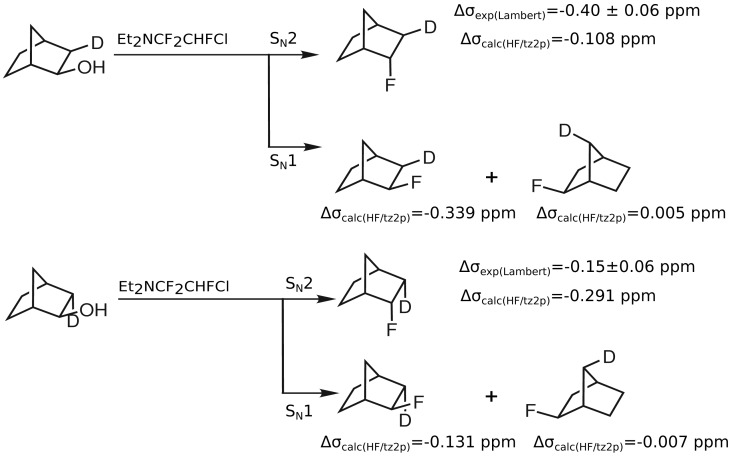
Computed ^19^F secondary isotope shifts of proposed products occurring from S*_N_*1 and S*_N_*2 mechanisms in comparison to ^19^F secondary isotope shifts obtained by Lambert *et al.* [15].

As the synthesis pathway proposed by Lambert *et al.* may also proceed via a S*_N_*1 mechanism and lead to *exo*-2-fluoronorbornanes, the corresponding computed isotope effects are given in Table 3. Secondary isotope shifts on ^19^F shieldings of −0.339 ppm for *exo*-2-fluoro(*exo*-3-^2^H)norbornane and of 0.005 ppm for *exo*-2-fluoro(*syn*-7-^2^H)norbornane are computed. In the case of the *exo*-2-fluoro(*endo*-3-^2^H)norbornane, a secondary isotope shift on ^19^F of −0.131 ppm is obtained and in the case of *exo*-2-fluoro(*anti*-7-^2^H)norbornane, this shift amounts to −0.007 ppm. These computed secondary isotope shifts on ^19^F shieldings are in much better agreement with those observed by Lambert *et al.* In contrast to this, the computed values of the proposed *endo*-2-fluoronorbornanes differ significantly from the secondary isotope shifts of products of S*_N_*1 reactions. This discrepancy suggests that rather *exo*-2-fluoronorbornanes than *endo*-2-fluoronorbornanes result from the substitution of *endo*-2-norbornanols with Et_2_NCF_2_CHFCl (Figure 3).

We carried out the synthesis of *endo*-2-fluoronorbornanes using the pathway proposed by Lambert *et al.*, starting from *exo*-2-norbornanoles [16]. While details on the experimental work will be given elsewhere, preliminary results on the ^19^F and ^13^C secondary isotope shifts of the corresponding products are given in Table 3. These experimental values show good agreement with the calculated ones of the corresponding *exo*-2-fluoronorbornanes and agree less well with the values of the *endo*-2-fluoronorbornanes proposed by Lambert *et al.* Our further investigations led to an alternative synthesis pathway, where the 2-fluoronorbornanes were prepared from 2-norbornyl tosylates or the corresponding deuterated compounds by nucleophilic substitution via S*_N_*1 and S*_N_*2 reactions; details will be reported elsewhere [16]. For *endo*-2-fluoro(*anti*-7-^2^H)norbornane and *endo*-2-fluoro(*endo*-3-^2^H)norbornane, ^19^F and ^13^C secondary isotope shifts were measured and show good agreement with the calculated ones (Table 2), while the secondary isotope shift on the ^19^F chemical shielding of *endo*-2-fluoro(*endo*-3-^2^H)norbornane observed by Lambert *et al.* could not be confirmed.

Since a mixture of compounds with ^2^H in 7- and 3-positions exists after the synthesis, an assignment of the secondary isotope shifts to the corresponding compounds is difficult. As mentioned in Section 2, the uncertainties in the ^13^C secondary isotope shifts are smaller than those for ^19^F, and this allows a better assignment of experimental values to the resonating nuclei. Using the calculated isotope shifts leads to assignments given in Table 2 and Tables 3, although for some nuclei the assignment is not unique, as for example in the case of *exo*-2-fluoro(*endo*-3-^2^H)norbornane and *exo*-2-fluoro(*anti*-7-^2^H)norbornane for carbons 4 and 5.

## 4. Conclusions

In the present study, we demonstrate how calculations of secondary isotope shifts can be used in order to distinguish different stereoisomers. Benchmark calculations for fluoroethane show, that compared to CCSD(T) calculations, secondary isotope shifts obtained at the MP2 level of theory exhibit an error in the order of 5%. While at the HF level of theory using triple-zeta basis sets, errors can amount 25%, this accuracy is usually still sufficient to even identify different conformers, as the relative secondary isotope effects often differ by more than 50% and sometimes even by an order of magnitude. Therefore, quantum chemical calculations of the differences in secondary isotope shifts can be applied in order to reliably distinguish between various compounds.

An example, where such calculations can be used to resolve ambiguous results in the literature are measurements by Lambert *et al.*, who presented secondary isotope shifts of *endo*-2-fluoronorbornanes with deuterium in *exo*-3- and *endo*-3-positions, which are now convincingly explained by the corresponding effects in *exo*-2-fluoronorbornanes with deuterium labels at the 3- and 7-positions (Figure 3). Our recent experimental results contradict the assignments of Lambert *et al.* and suggest that the deviations are due to different products as a consequence of a different mechanism than the one assumed in the original work. Calculations of the secondary isotope shifts of all possible products exhibit deviations from the results of Lambert *et al.* that are far outside the error bars of the computations but in very good agreement with the preliminary experimental results [16].

## Figures and Tables

**Table 1.** Secondary isotope shifts of fluoroethane in ppm with deuterium in gauche and antiperiplanar conformation at different levels of theory for the anharmonic force field *k_rss_* and the chemical shifts *σ* and for different temperatures.

**Level for** *σ*	**HF/tz2p**		**HF/qz2p**		**MP2/tz2p**		**HF/tz2p**		**MP2/tz2p**		**MP2/qz2p**		**CCSD(T)/qz2p**	
**Level for** *k_rss_*	**HF/tz2p**		**HF/qz2p**		**HF/tz2p**		**MP2/cc-pVTZ**		**MP2/cc-pVTZ**		**MP2/cc−pVTZ**		**MP2/cc−pVTZ**	

**temperature**	**0K**	**300K**	**0K**	**300K**	**0K**	**300K**	**0K**	**300K**	**0K**	**300K**	**0K**	**300K**	**0K**	**300K**
anti														
^19^F *^a^*	−0.417	−0.354	−0.446	−0.382	−0.437	−0.368	−0.461	−0.384	−0.483	−0.400	−0.513	−0.430	−0.493	−0.411
^13^C_1_	−0.004	−0.011	−0.003	−0.010	−0.008	−0.015	−0.005	−0.012	−0.010	−0.016	−0.009	−0.016	−0.008	−0.015
^13^C_2_	−0.266	−0.267	−0.265	−0.266	−0.240	−0.240	−0.267	−0.269	−0.242	−0.242	−0.240	−0.242	−0.253	−0.254
gauche														
^19^F *^a^*	−0.142	−0.115	−0.156	−0.130	−0.142	−0.114	−0.157	−0.126	−0.156	−0.124	−0.180	−0.148	−0.191	−0.158
^13^C_1_	−0.033	−0.038	−0.030	−0.036	−0.039	−0.044	−0.036	−0.041	−0.043	−0.047	−0.041	−0.045	−0.041	−0.045
^13^C_2_	−0.269	−0.270	−0.267	−0.268	−0.248	−0.248	−0.270	−0.271	−0.249	−0.250	−0.246	−0.248	−0.262	−0.263

Δ anti-gauche														
^19^F	−0.275	−0.239	−0.290	−0.252	−0.295	−0.254	−0.304	−0.258	−0.327	−0.276	−0.333	−0.282	−0.302	−0.253
^13^C_1_	0.029	0.027	0.027	0.026	0.031	0.029	0.031	0.029	0.033	0.031	0.032	0.029	0.033	0.030
^13^C_2_	0.003	0.003	0.002	0.002	0.008	0.008	0.003	0.002	0.007	0.008	0.006	0.006	0.009	0.009

a The experimental ^19^F isotope shift is −0.244 ppm [28]. The averaged isotope shift on CCSD(T) level is −0.240 ppm.

**Table 2.** Secondary isotope shifts of *endo*-2-fluoronorbornane in ppm at HF/tz2p level and for T = 300 K and experimental values with deuterium labels on different positions in the molecule.

	**Calculated (HF/tz2p)**			**Experimental values [32]**	

**atom**	***endo-3***	***exo-3***	***anti-7***	***endo-3***	***anti-7***
^13^C_1_	−0.013	−0.021	−0.095	0.000	−0.080
^13^C_2_	−0.006	−0.060	0.028	−0.026	0.022
^13^C_3_	−0.362	−0.367	−0.009	−0.387	0.000
^13^C_4_	−0.104	−0.094	−0.102	−0.102	−0.102
^13^C_5_	−0.037	−0.041	−0.022	−0.044	−0.027
^13^C_6_	0.004	0.004	−0.024	−0.005	−0.032
^13^C_7_	−0.022	−0.011	−0.344	−0.030	−0.341
^19^F	−0.291	−0.108	−0.049	−0.359	−0.064

**Table 3.** Secondary isotope shifts of *exo*-2-fluoronorbornane in ppm at HF/tz2p level and for T = 300 K and experimental values with deuterium labels on different positions in the molecule.

	**Calculated (HF/tz2p)**					**Experimental values [32]**		

**atom**	***endo-2***	***exo-3***	***endo-3***	***anti-7***	***syn-7***	***endo-2***	***endo-3***	***anti-7***
^13^C_1_	−0.113	−0.006	0.008	−0.087	−0.082	−0.112	0.007	−0.088
^13^C_2_	−0.385	−0.032	−0.038	0.016	0.001	−0.408	−0.038	0.021
^13^C_3_	−0.112	−0.328	−0.359	0.003	−0.027	−0.115	−0.350	0.000
^13^C_4_	0.010	−0.086	−0.101	−0.089	−0.086	0.010	−0.101	−0.090
^13^C_5_	0.001	−0.038	−0.040	−0.022	0.008	0.000	−0.050	−0.028
^13^C_6_	−0.020	0.003	0.001	−0.023	−0.014	−0.024	0.000	−0.028
^13^C_7_	0.013	−0.013	0.005	−0.355	−0.315	0.010	0.008	−0.349
^19^F	−0.794	−0.339	−0.131	−0.007	0.005	−0.799	−0.156	−0.008
